# Monitoring of cerebral oxygen saturation during resuscitation in out-of-hospital cardiac arrest: a feasibility study in a physician staffed emergency medical system

**DOI:** 10.1186/s13049-014-0058-y

**Published:** 2014-10-05

**Authors:** Jens-Christian Schewe, Marcus O Thudium, Jochen Kappler, Folkert Steinhagen, Lars Eichhorn, Felix Erdfelder, Ulrich Heister, Richard Ellerkmann

**Affiliations:** Department of Anaesthesiology, University of Bonn Medical Center, Sigmund-Freud-Str. 25, 53105 Bonn, Germany

**Keywords:** NIRS, CPR, Cerebral oximetry, Near infrared spectroscopy, Resuscitation, Mechanical chest compression

## Abstract

**Background:**

Despite recent advances in resuscitation algorithms, neurological injury after cardiac arrest due to cerebral ischemia and reperfusion is one of the reasons for poor neurological outcome. There is currently no adequate means of measuring cerebral perfusion during cardiac arrest. It was the aim of this study to investigate the feasibility of measuring near infrared spectroscopy (NIRS) as a potential surrogate parameter for cerebral perfusion in patients with out-of-hospital resuscitations in a physician-staffed emergency medical service.

**Methods:**

An emergency physician responding to out-of-hospital emergencies was equipped with a NONIN cerebral oximetry device. Cerebral oximetry values (rSO_2_) were continuously recorded during resuscitation and transport. Feasibility was defined as >80% of total achieved recording time in relation to intended recording time.

**Results:**

10 patients were prospectively enrolled. In 89.8% of total recording time, rSO_2_ values could be recorded (213 minutes and 20 seconds), thus meeting feasibility criteria. 3 patients experienced return of spontaneous circulation (ROSC). rSO_2_ during manual cardiopulmonary resuscitation (CPR) was lower in patients who did not experience ROSC compared to the 3 patients with ROSC (31.6%, ± 7.4 versus 37.2% ± 17.0). ROSC was associated with an increase in rSO_2_. Decrease of rSO_2_ indicated occurrence of re-arrest in 2 patients. In 2 patients a mechanical chest compression device was used. rSO_2_ values during mechanical compression were increased by 12.7% and 19.1% compared to manual compression.

**Conclusions:**

NIRS monitoring is feasible during resuscitation of patients with out-of-hospital cardiac arrest and can be a useful tool during resuscitation, leading to an earlier detection of ROSC and re-arrest. Higher initial rSO_2_ values during CPR seem to be associated with the occurrence of ROSC. The use of mechanical chest compression devices might result in higher rSO_2_. These findings need to be confirmed by larger studies.

## Background

Spontaneous circulation in out of hospital cardiac arrest (OHCA) may be restored in up to 50% of patients in the presence of well-trained emergency physicians [1-4]. Despite these promising results in the treatment of OHCA, survival rates remain low.

Discharge rates of 14% and 20% are reported in these emergency medical systems (EMS) [4], and 1-year survival rates can reach up to 11% [2]. Outcome depends on professional EMS treatment including proper post resuscitation therapy and implementation of treatment strategies as published in current guidelines [5]. Lately, investigations revealed an improvement in neurological outcome in patients treated with therapeutic hypothermia following return of spontaneous circulation (ROSC) after OHCA [6]. Multiple mechanisms are discussed to be responsible for this neuroprotective effect [7]. Undoubtfully, sufficient perfusion pressure during CPR is also crucial for neurological outcome. During basic life support chest compressions (frequency and depth of compression) as well as ventilation (end-expiratory CO_2_) are poor measures to evaluate the performance of adequate resuscitation. During CPR, cerebral blood flow decreases to 20-50% compared to normal values [8-10]. At the same time, neurological outcome and survival depend on sufficient cerebral blood flow. To date, it is impossible to provide helpful measurements to predict neurological outcome during out-of-hospital resuscitation.

Regional cerebral oximetry with near infrared spectroscopy (NIRS) has emerged as a surrogate parameter monitoring cerebral perfusion in the intraoperative and intensive care setting [11]. The method uses the effect that light in the near-infrared spectrum can penetrate the skull, thus allowing measurements of oxyhemoglobin. The absorption of light permits the measurement of oxyhemoglobin, desoxyhemoglobin, and total hemoglobin [12]. Ono et al. have revealed that decreasing intraoperative rSO_2_ values due to hypotension are associated with major morbidity and mortality after cardiac surgery [13]. Murkin et al. could show that intraoperative treatment of low rSO_2_ values resulted in decreased major organ morbidity or mortality [14]. Small clinical studies have addressed the feasibility of NIRS technology in measuring cerebral oxygen saturation in patients during CPR. These studies included in-hospital resuscitation [15-17], and out-of-hospital cardiac arrest [18,19], as well as a combination of the two [20,21]. The authors suggest that the use of NIRS for cerebral oximetry is promising for monitoring patients with cardiac arrest. However, none of these studies focused on routine use in a physician-staffed EMS. In summary, data is still limited, but available studies suggest that NIRS monitoring can provide a real-time non-invasive marker of cerebral perfusion and thus cerebral oximetry may have a role in optimising cerebral perfusion in cardiac arrest.

The aim of this study was to investigate the feasibility of NIRS monitoring in out-of-hospital cardiac arrests in a physician-staffed emergency medical service in daily routine. Cerebral oximetry (rSO_2_) was measured during standard cardiopulmonary resuscitation. This included the optional use of a mechanical chest compression device, a decision left to the discretion of the emergency physician.

## Methods

### Setting

The emergency medical service (EMS) of the City of Bonn serves 320,000 residents in a service area of 141 km^2^, with a population density of 2,250 persons/km^2^. The city reflects urban features. A total of 12 basic life support (BLS) units and 3 advanced life support (ALS) units serve (2 of them 24 hours/7 days and 1 only 10 hours weekdays) in a two tier system. The EMS has 84,240 BLS-unit hours/year and 19.410 ALS-unit hours/year.

The EMS system responds to 33,600 emergency calls, increasing about 3-5% per year, with 26,000 BLS-unit transports and 7,600 ALS unit interventions per year. In Bonn patients are admitted to 12 hospitals, all with different care levels.

The BLS units are at least staffed with one emergency medical technician (EMT) and one paramedic. The ALS units are staffed with one paramedic and one physician. Physicians on the ALS units are predominantly anaesthesiologists and have passed at least 2 years of postgraduate training as well as a special emergency training course according to regulations of the German medical academy. The incidence of CPR in the EMS of Bonn is within 45–55 CPRs/100,000 inhabitants/year.

### NIRS device

The NONIN EQUANOX Model 7600 regional oximetry system (Nonin Medical, Plymouth, Minnesota, USA) is a portable 4-wavelength cerebral oximeter. It weighs approximately 900 grams, plus 180 grams for 2 sensor pods in the 2 sensor configuration. Dimensions are 305×108×130 mm. Once the device is activated and a sensor is attached, it displays continuous rSO_2_ data in percent from 0 to 100. Readings are sampled every 4 seconds. The monitor is equipped with a storage battery lasting for 4 hours when fully charged.

### Study population/data acquisition

The study was performed from October 2012 to July 2013. One ALS unit, which is on duty from Monday until Friday from 8 a.m.- 6 p.m., was equipped with the NIRS device. Prior to the beginning of the study, 3 physicians were trained for half an hour in the use of the device according to legal requirements. Whenever dispatched to emergencies with suspected cardiac arrest, which was the case when a lifeless person was reported, the physician carried the NIRS device to the scene. All patients age >18 years with non-traumatic cardiac arrest were included. Exclusion criteria were: trauma, hypothermia <35°C, and pregnancy.

If the inclusion criteria were met, the physician immediately initiated the NIRS monitoring at the time of beginning of ALS. One optode was attached on the patient’s left forehead lateral of the midline and above the eyebrow to acquire the spectrum of the left frontal cortex. This process took less than 30 seconds and did not interrupt BLS. Time markers were set on the device for important events such as ROSC, use of mechanical compression devices, or termination of CPR. Advanced cardiac life support (ACLS) was performed according to 2010 ERC guidelines [5]. This included intubation by the emergency physician during ALS verified by direct laryngoscopy, auscultation and end-tidal CO_2_ measurement. Ventilator settings were adjusted according to the patient’s estimated body weight. The rSO_2_ value on the device monitor was covered for the study, so that the resuscitation team was blinded to rSO_2_ readings and the process of routine resuscitation was not altered by the NIRS device. Intended recording time of rSO_2_ started immediately after the emergency physician arrived at the scene with initiation of ALS and ended when the patient arrived at the emergency department or when CPR was terminated and death testfied. Data was transferred onto a computer via bluetooth and data was analyzed offline on a standard Excel spreadsheet also used for descriptive statistics. We defined feasibility as percentage (>80%) of total achieved recording time in relation to intended recording time. Data is displayed as mean ± standard deviation if not mentioned otherwise.

The study was approved by the local ethics committee of the University of Bonn Medical Center.

## Results

Ten consecutive patients (8 male and 2 female) were included. Mean age was 73 ± 13 years ranging from 50 to 90 years (see Table 1 for demographic data). Overall recording time was 237 minutes and 36 seconds. The oximetry signal was lost during 24 minutes and 16 seconds, resulting in 213 minutes and 20 seconds of clinical recording time during CPR. With 89.8% total recording time, feasibility criteria were met.

**Table 1:** **Demographic and NIRS data of all 10 included patients**

**Patient**	**Age**	**Gender**	**Initial ECG**	**rSO**_**2**_ **during manual CPR (%)**	**rSO**_**2**_ **during mechanical compression (%)**	**rSO**_**2**_ **after ROSC (%)**	**rSO**_**2**_ **after end of CPR (no ROSC, %)**
1	64	**♂**	VF	35.3 ± 6.9	-	58.3 ± 4.6	-
2	77	**♂**	Asy	35.4 ± 3.0	-	-	33.1 ± 0.9
3	82	**♂**	Asy	30.5 ± 0.7	-	-	30.9 ± 0.3
4	90	**♂**	Asy	35.6 ± 1.7	-	-	36.0 ± 0.4
5	53	**♂**	VF	45.7 ± 5.3	-	63.2 ± 8.0	-
6	76	**♂**	Asy	38.0 ± 5.9	-	-	29.3 ± 1.4
7	82	**♀**	Asy	18.2 ± 3.1	-	-	20.4 ± 0.5
8	74	**♂**	Asy	30.5 ± 1.3	-	35.3 ± 4.2	-
9	50	**♂**	VF	37.4 ± 4.7	42.2 ± 2.9	-	-
10	84	**♀**	Asy	21.3 ± 2.5	25.4 ± 2.3	-	-

Data is presented in mean ± standard deviation. VF: ventricular fibrillation, Asy: asystole.

**Figure 1:**
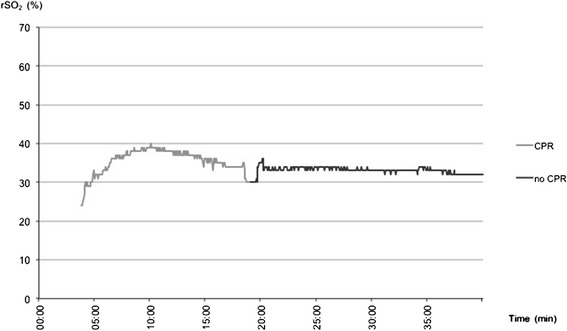
**NIRS data of patient #2 who never experienced ROSC.**

ROSC was achieved in 3 of 10 patients (30%). Figure 1 displays data of a patient having not experienced ROSC. Initially 3 patients had ventricular fibrillation (VF) on arrival at the scene, while 7 patients were asystolic. Of the 3 patients with VF, 2 experienced ROSC. Of the 7 patients with initial asystole, 1 could be converted into a shockable rhythm and ROSC was established following defibrillation.

**Figure 2:**
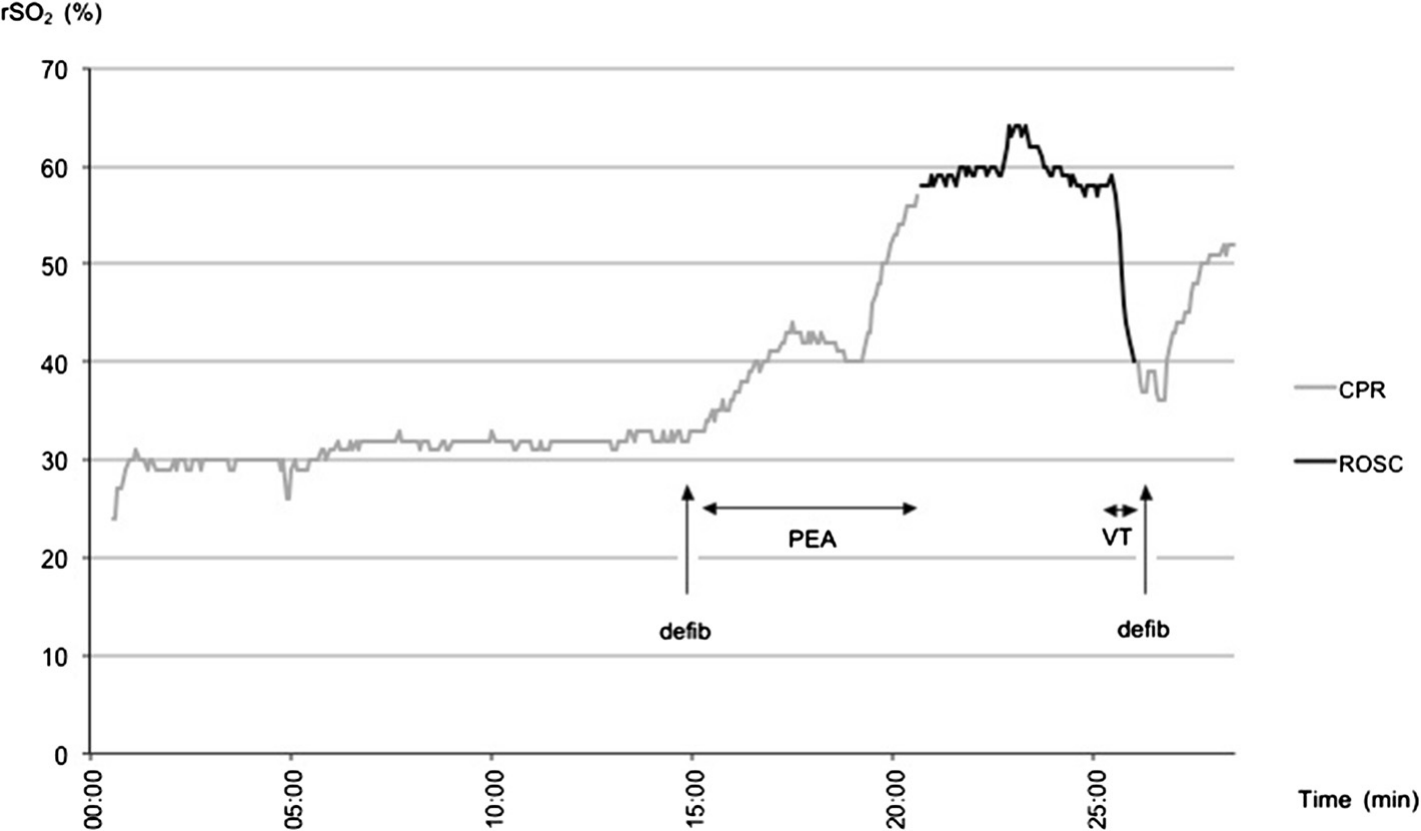
**NIRS data of patient** **#1 who had initial VF and experienced ROSC.** After defibrillation and conversion into PEA there was an increase in rSO_2_ before ROSC could be diagnosed. rSO_2_ decreased again prior to re-arrest.

**Figure 3:**
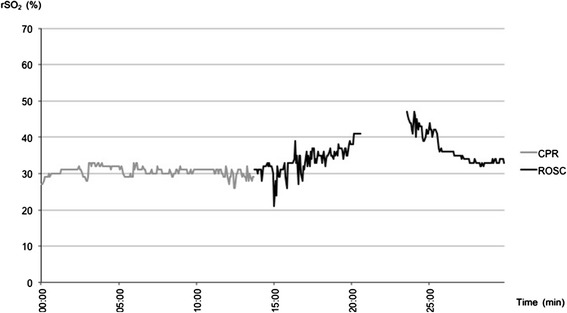
**NIRS data of patient #8 with ROSC after initial asystole.** After ROSC rSO_2_ increased slowly while circulation could only be maintained with high doses of vasopressors.

All 3 patients with ROSC were admitted to hospital. Of these patients, one (patient #1) was admitted to ICU after coronary intervention but eventually showed no signs of neurological recovery. CT scans showed extensive brain damage, so that therapy was limited and the patient died on ICU (see Figure 2 for NIRS data). The second patient (patient #5) to experience ROSC was discharged after successful coronary intervention and ICU stay without neurological impairment (Cerebral Performance Category, CPC 1, good performance). In the third patient where ROSC was achieved despite initial asystole (patient #8), rSO_2_ increased slowly after ROSC was detected (Figure 3). After ROSC, spontaneous circulation could only be maintained under massive doses of vasopressors during transport into the hospital and the patient died shortly after admission. 2 patients were transported to a hospital with ongoing CPR with manual as well as mechanical chest compressions (load distributing band CPR, AutoPulse® device, ZOLL, Chelmsford, MA, USA). In both cases, CPR attempts were terminated in the emergency department of the admitting hospital, based on further examination and laboratory results by the hospital physicians. In 5 cases (50%), resuscitation was unsuccessful and the patients died at the scene.

**Figure 4:**
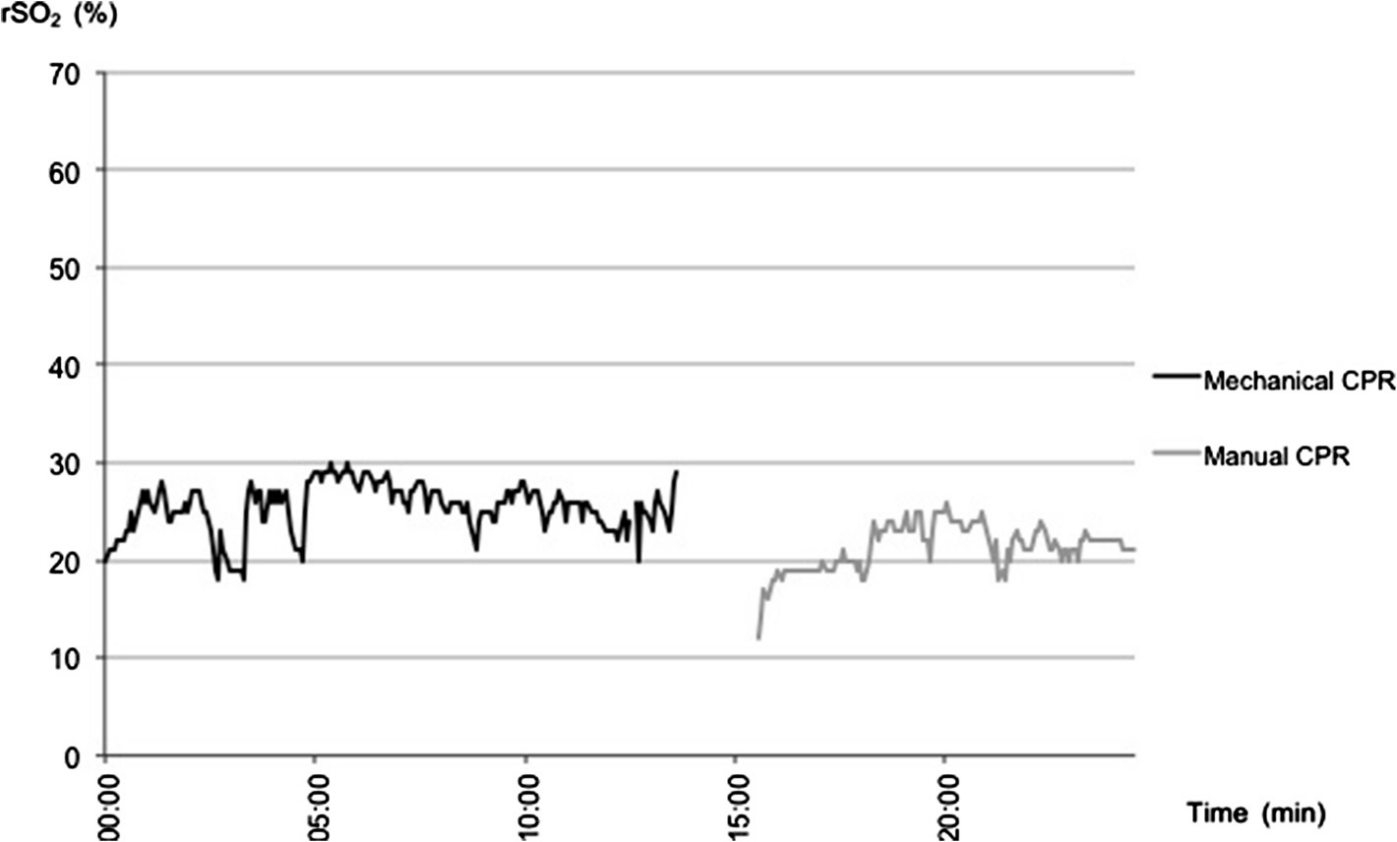
**NIRS data of patient #10 who never experienced ROSC.** A mechanical chest compression device (load distributing band CPR) was used for CPR. Due to limited battery capacity CPR had to be continued manually resulting in lower rSO_2_ values.

Mean rSO_2_ under manual chest compression (n = 10) was 32.8% ± 8.1 compared to 29.9% ± 5.9 after termination of chest compressions (n = 7) (relative decrease in rSO_2_ of 8.7%). A mechanical chest compression device was used in 2 patients. Since battery capacity of the device was limited and CPR had to be continued manually, rSO_2_ readings during manual and mechanical chest compression could be recorded. In one patient, mechanical compression led to a relative increase in rSO_2_ of 12.7% (rSO_2_ 42.2% vs. 37.4%). In the second patient rSO_2_ during mechanical compression was 19.1% higher compared to manual compression (rSO_2_ 25.3% vs. 21.3%, see Figure 4).

In all patients with ROSC (n = 3), an increase in rSO_2_ was observed under spontaneous circulation. Mean rSO_2_ increased from 37.2% ± 7.7 before ROSC to 52.3% ± 14.9 at time of ROSC, a relative increase of 40.6%.

Offline analysis showed that ROSC was indicated by an increase in rSO_2_ well before the emergency physician diagnosed ROSC through a palpable pulse in 2 patients (Figure 2). In 1 patient with initial asystole rSO_2_ increased after ROSC was diagnosed (Figure 3). In parallel, rSO_2_ decreased before the emergency physisican decided to restart CPR due to recurrent ventricular fibrillation in 2 patients (see raw data of patient 1, Figure 2).

**Figure 5:**
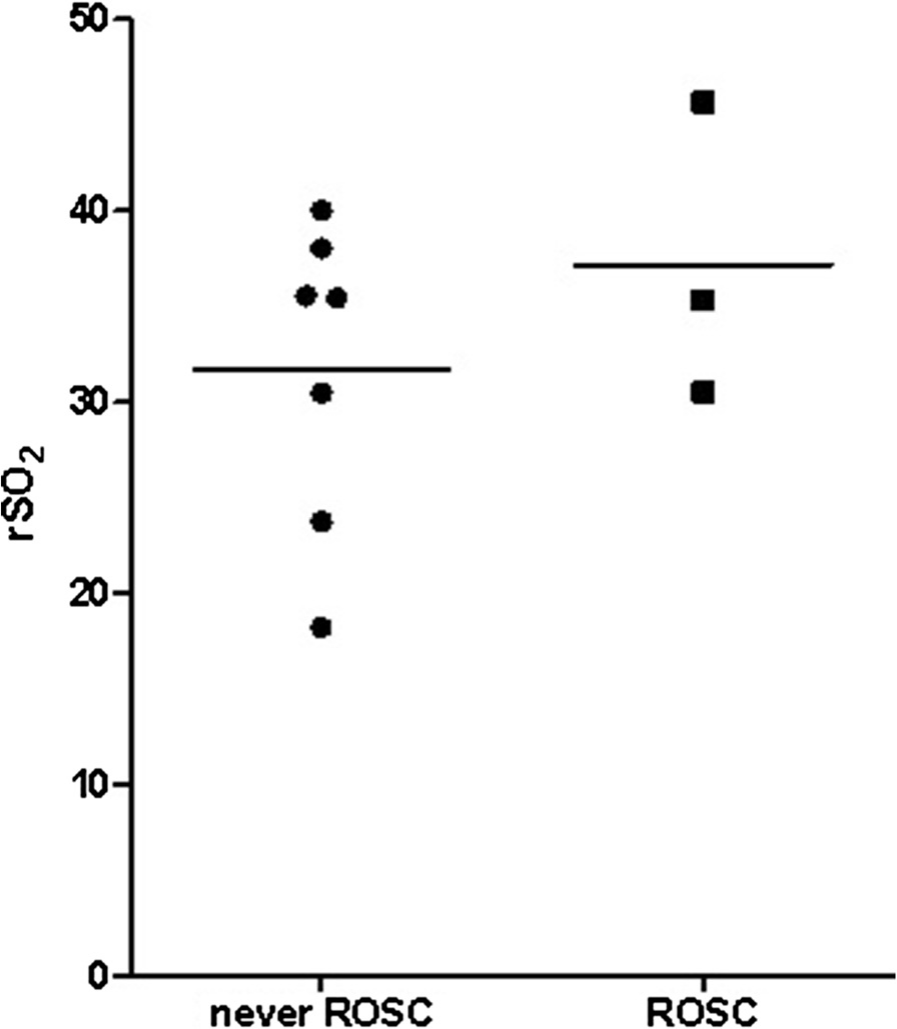
**Scatter plot and mean rSO**_**2**_ **values during CPR of patients (n = 7) who never experienced ROSC and patients (n = 3) with ROSC.**

rSO_2_ during CPR was lower in patients who did not experience ROSC compared to the 3 patients with ROSC (31.6 ± 7.4%, vs. 37.2 ± 17.0%, see Figure 5).

## Discussion

This study demonstrated the feasibility of rSO_2_ monitoring via NIRS during resuscitation in out-of-hospital cardiac arrest in a physician-staffed emergency medical system, both at the scene and also during transport of patients. Feasibility criteria for monitoring rSO_2_ were met by the NIRS device, providing values in 89.8% of total recording time. Moreover, a concomitant detection of increasing NIRS values in patients where ROSC was obtained and a decrease of NIRS values following re-arrest with the onset of VT was observed.

In a different study, Newman et al. reported no detectable rSO_2_ signal in patients with out-of-hospital cardiac arrest [18]. Contrary to these findings, we could record stable values, possibly due to a general technological improvement in modern NIRS devices.

Only 1 out of 10 patients survived without neurological impairment (Cerebral Performance Category, CPC 1, good performance). This conforms with the general resuscitation outcome of the EMS of the City of Bonn as reported in a larger study [4]. It is remarkable that this patient had the highest rSO_2_ readings during CPR and ROSC (45.7% ± 5.27 and 63.2%, ± 8.02, respectively). However, this individual finding is too limited to draw conclusions on general neurological outcome and NIRS measurements during CPR.

Ahn et al. suggested a relationship between rSO_2_ and ROSC as well as a relationship between low rSO_2_ and unsuccessful CPR [21] Koyama et al. drew similar conclusions following their results [17]. Our results are consistent with these previous findings, observing lower rSO_2_ readings corresponding with futile resuscitation efforts compared to rSO_2_ values in patients with successful CPR (rSO_2_ 31.5% ± 7.1 vs. 37.2% ± 17.0, Figure 5). The second patient to experience ROSC survived more than 24 hours, but ultimately suffered hypoxic brain damage. In this patient, ROSC was detected after a notable increase in rSO_2_ (35.3%, ± 6.94 during CPR vs. 58.3%, ± 4.63 after ROSC). However, before ROSC occurred, NIRS readings showed a long period of low rSO_2_ (Figure 2). One could speculate rSO_2_ levels were low for too long and neurological damage was therefore irreversible. Unfortunately, NIRS monitoring was not continued after hospital admission. In future, the initial NIRS reading and the trend during the initial phase of resuscitation might help to predict outcome in terms of ROSC, survival and/or neurological outcome.

In the 2 patients mentioned above, rSO_2_ decreased before cardiac re-arrest was observed by the emergency physician. Frisch et al. published similar observations for peripheral tissue oximetry [19] and Meex et al. for cerebral oximetry [20]. We believe that NIRS could potentially improve CPR protocols by serving as an early-warning system detecting hemodynamic instability after ROSC prior to re-arrest.

In both of these patients, NIRS values increased rapidly with ROSC. In contrast, rSO_2_ increased slowly after ROSC in patient 8 (see Figure 3). Vasopressors had to be used to sustain circulation during transportation until therapy was finally withdrawn in the emergency department. Our study is too limited to draw assumptions between the dynamics of increase in NIRS following ROSC and stable versus unstable hemodynamics and the possible underlying pathology.

Several studies have found an increase in rSO_2_ after ROSC in patients [18-20]. Only Kämärainen et al. reported that rSO_2_ increases prior to clinical detection of ROSC [15]. It may be speculated that the time delay between increase of rSO_2_ in 2 cases of our study prior to documented ROSC is due to delayed clinical detection of ROSC by the physician. Guidelines recommend continuous chest compression for 2 minutes after defibrillation before analysis of cardiac rhythm and pulse. Nevertheless, within these two minutes, ROSC might be detected by increasing rSO_2_. In future, it may be possible to differentiate between sufficient and insufficient cerebral perfusion as detected by a significant increase in NIRS guiding the physician to continue or interrupt manual chest compression.

The use of mechanical compression devices for CPR still remains controversial. In previous studies, a higher arterial pressure and cardiac perfusion pressure were obtained with mechanical CPR devices versus manual chest compression [22,23]. A recent meta-analysis by Westfall et al. suggests the superiority of mechanical devices versus manual chest compression regarding ROSC [24]. However, there is insufficient evidence for the superiority of mechanical chest compression devices in terms of neurological outcome [25]. To our knowledge, we are the first to provide data on NIRS monitoring during mechanical and manual chest compression in OHCA. Kämärainen et al. could not show a significant increase in rSO_2_ by improving CPR with a feedback device (improved quality of manual chest compression) [15]. Whether this also applies to mechanical CPR devices has not been shown. In both patients where the device was applied, rSO_2_ values were increased by 12% and 19% compared to manual compression. This finding could support the theory that the use of mechanical CPR devices can achieve higher cerebral perfusion pressure compared to manual chest compression. However, we cannot rule out that increasing arterial CO_2_ might have influenced measured rSO_2_ values based on autoregulation of cerebral blood flow influenced by changing arterial CO_2_. The detected increase in measured rSO_2_ values during mechanical chest compressions could in theory also be explained by increasing arterial CO_2_ [26].

During resuscitation, end-tidal CO_2_ values are also used as a surrogate parameter for arterial CO_2_ and thus as an indirect measurement of the achieved cardiac output during CPR. End-tidal CO_2_ during resuscitation is believed to be a quality marker of CPR performance [27]. In this study, end-tidal CO_2_ values during resuscitation were measured, but not recorded to the memory card due to technical reasons, and thus were not available for offline analysis, which limits our observational study.

## Conclusions

Cerebral oximetry using NIRS for out-of-hospital cardiac arrest is feasible in a physician-staffed EMS. rSO_2_ monitoring can help detect ROSC and hemodynamic instabilities resulting in re-arrests. rSO_2_ values measured during application of a mechanical chest compression device were higher than during manual chest compression. Further investigations are needed to confirm whether these results can be explained by improved cerebral perfusion.
